# Concordance between the Different Cardiovascular Risk Scores in People with Rheumatoid Arthritis and Psoriasis Arthritis

**DOI:** 10.1155/2019/7689208

**Published:** 2019-03-14

**Authors:** Cristina Gonzalez-Martin, Silvia Grande Morais, Sonia Pertega-Diaz, Teresa Seoane-Pillado, Vanesa Balboa-Barreiro, Raquel Veiga-Seijo

**Affiliations:** ^1^Clinical Epidemiology and Biostatistics Research Group, Instituto de Investigación Biomédica de A Coruña (INIBIC), Complexo Hospitalario Universitario de A Coruña (CHUAC), SERGAS, Universidade da Coruña, Hotel de Pacientes 7^a^ Planta, C/As Xubias de Arriba, 84, 15006 A Coruña, Spain; ^2^Nurse Center of Saude Carballeira, Orense, Spain

## Abstract

**Aim:**

To determine the cardiovascular risk and the concordance between the different scores in people with rheumatoid arthritis (RA) and psoriatic arthritis (PsA).

**Methods:**

Observational descriptive study of prevalence. Performed in the Rheumatology Service and the Clinical Epidemiology and Biostatistics Unit of the University Hospital Complex of A Coruña (Spain). Patients diagnosed with RA or PsA, older than 18 years of age were included. Measurements: sociodemographic, anthropometric variables of the disease, comorbidity, cardiovascular risk, and therapeutic management.

**Results:**

151 subjects (75 RA and 76 PsA) were studied. The average age was 57.9 ± 12.2 years, 61.6% being women. The average of the Charlson index was 2.8 ± 1.5. 43% were overweight. 46.5% were classified as cardiovascular risk, and the average percentage was 33.3% by Framingham. The best agreement has been between Framingham and Dorica (*k* = 0.709; *p* < 0.001), classifying more than 80% of the cases in the same risk categories.

**Conclusions:**

The most prevalent risk factors were overweight and obesity, followed by smoking and hypertension. The prevalence of patients with moderate/high cardiovascular risk varies according to the score used, the levels of concordance being the scores of Framingham and Dorica.

## 1. Introduction

Rheumatoid arthritis (RA) and psoriatic arthritis (PsA) are two of the most prevalent rheumatic diseases which have an impact on the health system [1]. Among the repercussions of these diseases, their relationship with an increase in cardiovascular disease (CVD) [2, 3], morbidity, and mortality [1] stands out, owing to the role played by inflammation in both pathologies [4]. Thus, it has been observed that the CVD condition is the main cause of death, both in people with RA and in people with PsA [5, 6]. In the case of RA, life expectancy has been reduced from 3 to 10 years compared with the general population [7]. The cardiovascular risk (CVR) factors described in the literature are cholesterol, diabetes, hypertension, genetic inheritance, stress, tobacco, physical inactivity, obesity, and heart rate [8]. The CVD establishes the probability of suffering in a given period of time, generally 5 or 10 years, a cardiovascular episode [9]. The scientific literature reveals that the risk factors described and attributed to RA are hypertension, dyslipidemia [3], physical inactivity, obesity, and diabetes [10, 11], defined as highly prevalent in this population [12]. These factors can be fed back by the disease itself, as in the case of pain, which contributes to physical inactivity and obesity. Furthermore, it has been seen that the increase in CVR in people with RA has an effect similar in magnitude to that of diabetes [13]. A higher prevalence of heart failure [14, 15] and of acute myocardial infarction (41–68%) has also been found with respect to the general population [16, 17].

People with PsA [18] have more CVR factors than people with RA [19], constituting one of the most relevant causes of death [6].

In this way, the magnitude of the problem induces the interest of studying the recognized increase in CVR in these diseases [5], if it is also taken into account that it constitutes the main cause of death in these populations [20].

Therefore, the aim of the present research was to determine the CVR and the concordance between the different scores in people with RA and PsA.

## 2. Methods

An observational descriptive study of prevalence was carried out in the Rheumatology Service and in the Clinical Epidemiology and Biostatistics Unit of the University Hospital Complex of A Coruña (CHUAC) (A Coruña, Spain). Patients diagnosed with RA or PsA were included in the study who went consecutively to the Rheumatology Service during the study period (December 2016 to July 2017) and who gave their written informed consent to participate in the study. The study was approved by the Clinical Research Ethics Committee of Galicia (CEIC 2016/544). The following variables were studied for each person included in the research: sociodemographic variables (age, sex, family history, smoking habit, level of studies, work situation, and type of activity); anthropometric variables (body mass index (BMI)); abdominal circumference (cm), hip (cm), and neck (cm); waist circumference index (WCI) (cm); waist height index (WHI) (cm)); disease variables (type of time evolution of the disease); comorbidity variables (Charlson comorbidity index [21], systolic blood pressure (SBP), diastolic blood pressure (DBP), ankle-brachial index (ABI), total cholesterol level, HDL-cholesterol, left ventricular hypertrophy, and diabetes mellitus); cardiovascular risk (Framingham–Wilson, Score, Dorica, and Regicor) [22]; and therapeutic management variables (medication at the time of the interview). An electrocardiogram was performed on all participants in the study. The data were obtained from the examination and the clinical history and through the analytical parameters.

To perform the ABI, the patient was placed in the supine position. A cuff was used for the arm and another for the ankle of at least 40% of the circumference of the limb. The sleeve should be clean and dry. Doppler tube from 8 to 10 MHz was used. The blood pressure was taken simultaneously in the 2 arms with the validated automatic device Microlife WATCH BP OFFICCE ABI®, which determines differences between the 2 arms, making 3 measurements separated by one minute each, and making, the same device, an average of the 3 measurements in each arm. The device is validated for patients with atrial fibrillation and to perform the ABI automatically. It was measured in the posterior tibial artery (in the lower limbs) and the cuff was placed above the malleoli. ABIs with values <0.99 and ≥1.30 were considered pathological. The process was always performed by the same nurse in all patients.

To perform the electrocardiogram, the patient was placed on the stretcher, with the thorax, ankles, and wrists exposed, with arms and legs separated from the body. The corresponding electrodes were placed. The speed of the paper was adjusted to 25 mm/second and the tension to 10 mm/mv, and the way to do it in automatic mode was selected. The PageWriter TC20/TC30 electrocardiograph was used.

To avoid the variability of these measuring instruments, the recommendations of the manufacturers were followed. The devices were checked every 6 months. The measurements were always carried out by the same nurse, using the same procedure.

A descriptive study of the variables was carried out. The comparison of means between two groups was carried out using the Student's *t*-test or the Mann–Whitney test as appropriate after verification of normality with the Kolmogorov–Smirnov test. The association of qualitative variables was determined with the chi-square or Fisher's test as appropriate. The *p* value <0.05 has been considered as statistically significant. The concordance between the different scores was analyzed through the kappa concordance index.

## 3. Results

The general and comorbidity characteristics are shown in Table 1. The mean age at the time of the interview was 57.9 ± 12.2 years, 61.6% being women. The mean evolution of the disease was 9.2 ± 7.6 years, and the Charlson comorbidity index was 2.8 ± 1.5. In terms of BMI, a high percentage of overweight (43%) and obesity (28.5%) was observed. 19.9% declared to be a smoker and 31.8% ex-smoker. 71.5% did not present a family history of the study pathologies (RA and PsA).

The people diagnosed with RA showed greater age at diagnosis (51.1 ± 12.2 vs 46.4 ± 13, *p*=0.024), predominance of the female sex (77.3% vs 46.1%, *p* < 0.001), and of basic studies (64% vs 45.3%, *p*=0.022). Both groups showed similar BMI and comorbidity index. In contrast, a higher Charlson index adjusted for age was observed in patients with RA (3.1 ± 1.5 vs 2.5 ± 1.4, *p*=0.020).

Table 2 shows the CVR of the sample studied and its factors. The most prevalent pathology in our sample was hypertension (39.6%), followed by diabetes (10.3%), 8.1% being without target organ damage. Ventricular hypertrophy was present in 4.6% of cases. As for various anthropometric measurements, the mean of the WCI was 0.9 ± 0.1 and that of the WHI of 0.6 ± 0.1. None of these measurements showed statistically significant differences between the study pathologies. The patients presented total cholesterol of 205.2 ± 37.8 mg/dl, with mean values of LDL 122.1 ± 31.9 mg/dl and HDL 57.2 ± 15.4 mg/dl, showing no differences between the RA and PsA.

The mean of the ABI was 1.2 ± 0.1 and the heart rate in the sample studied was 71.3 ± 13.4, without finding significant differences between the two pathologies studied.  Table 3 shows the CVR classified according to the different scores as well as the concordance between them. Thus, it has been classified as moderate/high by 46.5% according to Score, by 33.3% according to Framingham, by 24.1% according to Dorica, and by 4.3% according to Regicor. A good concordance between Framingham and Dorica is observed (*k* = 0.709; *p* < 0.001), classifying more than 80% of the cases in the same risk categories. Also, the agreement was good between Framingham and Score (*k* = 0.464; *p* < 0.001), classifying more than 70% of the cases in the same risk categories. The characteristics and CVR factors were compared between the general population and rheumatic diseases in Table 4. The BMI observed in the general population is higher than that observed in patients with rheumatic disease, there being significant differences in the case of RA. (29.2 ± 4.7 vs. 27.7 ± 4.8, *p*=0.006). A lower comorbidity was observed in the general population, being significantly lower in relation to the present one in RA (2.2 ± 1.8 vs 3.1 ± 1.5, *p* < 0.001). The general population showed significantly higher LDL cholesterol values than those observed in patients with rheumatic disease. The most prevalent pathology in the general population was hypertension (36.5%), showing a similar percentage to that observed in RA (34.7%) and PsA (43.4%).

## 4. Discussion

In our study, the mean age of the patients at the time of diagnosis of arthritis was 48.7 years, with a predominance of females: data similar to those observed in the CARMA [23] project and a more recent study by Castañeda [24]. In the comparison between patients with RA and PsA, results were found in agreement with those obtained in the CARMA multicenter project, with a greater age at diagnosis in patients with RA, mostly women. In the literature, we find articles [20, 25–28] that deal with the topic of study, revealing the influence of cardiovascular diseases in rheumatic diseases, and thus exposing their importance. Research on CVR factors in PsA is scarcer than that in RA or with psoriasis [29].

The literature concludes the increase in CVR in these diseases, as well as in morbidity and mortality, constituting the systemic inflammation itself as an independent CVR factor [30–32], not forgetting the contribution of traditional risk factors (which can be modified by medication) [2, 26].

In general, the traditional factors of hypertension, diabetes, and obesity have a great association with RA and PsA (and this is also in case with hyperlipidemia) [32–34]. In relation to the BMI, we found high data of overweight (44.0%) and obesity (26.7%) in our article. Accordingly, Kitas and Gabriel [5] affirm that the relationship between BMI and mortality in people with RA is remarkable, due to the greater accumulation of body fat (known as rheumatoid cachexia) [35], unlike other investigations [33, 36] that claim to see that some patients have a low BMI associated with higher CVD mortality [30]. Likewise, no data have been found in other studies on the results of the different anthropometric data shown in this article (such as the waist–hip index), which is highly recommended [36], but it is noteworthy that abdominal fat is associated with insulin resistance, cardiometabolic risk, and the inflammatory load [5]. For its part, despite the scarcity of studies on body composition in PsA, it is reported that excess adiposity increases the CV [35] risk, being closely related to obesity [28], being associated with a greater metabolic risk than RA, and constituting obese BMI as a risk factor for psoriasis [37].

In our study, we found almost 20% of smokers; similar results in other researches (15.6%) [38]were observed. This smoking habit is associated with an increased risk of developing RA and increased activity of the disease [30]. In relation to hypertension, 35.6% RA and 43.4% PsA suffered it in the study sample. Lower percentages were objectified in other studies in RA (7.3%) [13]. Regarding PsA, Panoulas et al. [39] state that it is superior in patients with RA, ranging from 4 to 73%, being the cause of the sample sizes, the definition of hypertension, or the study population. Therefore, it is not clear that there is a higher prevalence of this pathology than in the RA or general population. More similar data with other investigations correspond to those of DM and RA (prevalence of 10%) [38], with a higher prevalence of this condition in PsA (23%) [31].

On the other hand, the literature shows inconsistent findings about lipid levels as the disease evolves, with a clear alteration of the lipid profile [35]. In our study, with an average time of evolution of the disease of 9.2 years, we obtain an average of total cholesterol of 208.6 and LDL 121.8. This LDL value is lower than that found in the general population, as in other investigations [40], which can be explained by the evolution of the disease [41] and questioning its clinical relevance in RA [40].

Reviewing the literature, Jurcut et al. [42] found an increased risk of myocardial infarction in people with RA. Regarding PsA, it is related to higher prevalences of heart diseases (heart failure or cerebrovascular disease) [29]. In line with the above, we have not found data to contrast our results on left ventricular hypertrophy in the study populations, as well as to relate the Charlson comorbidity score with the pathologies under study. It could be mentioned in this discussion that antirheumatic drugs, especially methotrexate and/or biologics, appear to reduce CVR by effectively decreasing systemic inflammation [41].

In relation to the CVD scores, Arts et al. [43], after reviewing the literature, state that the risk score of Framingham, Reynolds, and Score classify 60% of patients with RA as lower risk, agreeing with other investigations in which they underestimate CVR in RA [43]. Liao and Solomon [11] describe the need to develop scores that include inflammatory markers in addition to traditional cardiovascular risk factors so as not to underestimate CVR in these conditions [44, 45]. In our study, the scores show higher prevalences in a low CVR, which may be due to the time of evolution of the disease or the treatment received [43], since the events are more common within the first 7 years of the disease.

## 5. Conclusions

Cardiovascular risk factors have a great impact on people with RA and PsA. The most prevalent were overweight and obesity, followed by smoking and hypertension. The prevalence of moderate/high cardiovascular risk varies according to the score used, the Framingham and Dorica scores being the most concordant. The control of the disease in an integral way constitutes a fundamental field in the reduction of CVD risk in both diseases, fundamental for the quality of life of people with RA and PsA.

## Figures and Tables

**Table 1:** General characteristics and comorbidity of the global sample and according to the type of arthritis.

	Global, *n*=151	Rheumatoid arthritis, *n*=75 (49.7%)	Psoriatic arthritis, *n*=76 (50.3%)	
	Mean ± SD	Mean ± SD	Mean ± SD	*p*
Age dx (years)	48.7 ± 12.8	51.1 ± 12.2	46.4 ± 13	**0.024**
Age interview (years)	57.9 ± 12.2	60 ± 12.4	55.8 ± 11.8	**0.037**
BMI (kg/m^2^)	28 ± 5.1	27.7 ± 4.8	28.3 ± 5.3	0.445
Charlson index	1.4 ± 0.8	1.4 ± 0.8	1.3 ± 0.7	0.320
Adjusted Charlson index	2.8 ± 1.5	3.1 ± 1.5	2.5 ± 1.4	**0.020**
	*n* (%)	*n* (%)	*n* (%)	*p*
Sex				**<0.001**
Male	58 (38.4)	17 (22.7)	41 (53.9)	
Female	93 (61.6)	58 (77.3)	35 (46.1)	
Family history				**<0.001**
No	108 (71.5)	68 (90.7)	40 (52.6)	
Yes	43 (28.5)	7 (9.3)	36 (47.4)	
BMI (kg/m^2^)				0.910
Normal (BMI < 25)	41 (27.2)	20 (27.4)	21 (27.6)	
Overweight (25 ≥ BMI < 30)	65 (43)	33 (45.2)	32 (42.1)	
Obesity (BMI ≥ 30)	43 (28.5)	20 (27.4)	23 (30.3)	
Smoking habit				0.066
Nonsmoker	73 (48.3)	36 (48)	37 (48.7)	
Ex-smoker	48 (31.8)	29 (38.7)	19 (25)	
Smoker	30 (19.9)	10 (13.3)	20 (26.3)	
Studies				**0.022**
No/primary	82 (54.3)	48 (64)	34 (45.3)	
Superior	68 (45)	27 (36)	41 (54.7)	
Employment situation				0.148
Inactive	94 (62.3)	51 (68)	43 (56.6)	
Active	57 (37.7)	24 (32)	33 (43.4)	
Type of activity				0.509
Seated	18 (11.9)	7 (14.9)	11 (21.2)	
Standing/movement	51 (33.8)	27 (57.4)	24 (46.2)	
Mixed	30 (19.9)	13 (27.7)	17 (32.7)	
Medication				
Methotrexate	77 (56.6)	40 (57.1)	37 (56.1)	0.899

Age dx: age at diagnosis; BMI: body mass index.

**Table 2:** Cardiovascular risk factors and scores.

	Global, *n*=151	Rheumatoid arthritis, *n*=75 (49.7%)	Psoriatic arthritis, *n*=766 (50.3%)	
	Mean ± SD	Mean ± SD	Mean ± SD	*p*
Perimeter (cm)				
Abdominal	97.8 ± 13.8	97.6 ± 13.0	98.1 ± 14.7	0.823
Hip	102.4 ± 10.4	103.1 ± 10.2	101.8 ± 10.6	0.454
Neck	37.8 ± 4.5	37.8 ± 4.2	37.9 ± 4.7	0.861
WCI (cm)	0.9 ± 0.1	0.9 ± 0.1	0.9 ± 0.1	0.768
WHI (cm)	0.6 ± 0.1	0.6 ± 0.1	0.6 ± 0.1	0.423
Total cholesterol	205.2 ± 37.8	208.6 ± 40.7	201.9 ± 34.7	0.133
HDL	57.2 ± 15.4	59.0 ± 16.7	55.3 ± 13.8	0.158
LDL	122.1 ± 31.9	121.8 ± 33.7	122.5 ± 30.9	0.904
Ankle-brachial index	1.2 ± 0.1	1.2 ± 0.1	1.2 ± 0.1	0.929
Heart rate	71.3 ± 13.4	70.7 ± 14.5	71.8 ± 12.3	0.684
Cardiovascular risk scores				
Framingham	9.8 ± 8.6	9.6 ± 8.1	9.9 ± 9.1	0.807
Regicor	3.9 ± 3.1	3.7 ± 2.7	4.0 ± 3.6	0.640
Dorica	6.4 ± 5.9	5.9 ± 5.5	6.8 ± 6.3	0.386
Score	1.9 ± 2.2	2.1 ± 2.2	1.7 ± 2.1	0.281
	*n* (%)	*n* (%)	*n* (%)	*p*
LVH				0.276
No	144 (95.4)	70 (93.3)	74 (97.4)	
Yes	7 (4.6)	5 (6.7)	2 (2.6)	
Arterial hypertension				0.330
No	90 (60.4)	47 (64.4)	43 (56.6)	
Yes	59 (39.6)	26 (35.6)	33 (43.4)	
Charlson index				
PAD	1 (0.7)	1 (100.0)	0 (0.0)	0.515
Cerebrovascular disease	1 (0.7)	1 (100.0)	0 (0.0)	0.515
Heart failure	2 (1.5)	1 (50.0)	1 (50.0)	0.737
AMI	3 (2.2)	2 (66.7)	1 (33.3)	0.522
Diabetes	14 (10.3)	7 (50.0)	7 (50.0)	0.979
Without organ damage	11 (8.1)	5 (45.5)	6 (54.5)	0.459
With organ injuries	3 (2.2)	2 (66.7)	1 (33.3)	0.522

WCI: waist circumference index; WHI: waist height index; HDL: high-density lipoprotein; LDL: low-density lipoprotein; LVH: left ventricular hypertrophy; PAD: peripheral arterial disease; AMI: acute myocardial infarction.

**Table 3:** Classification of cardiovascular risk according to the different scores and concordance.

	Framingham	Dorica	Regicor	Score
	*n* (%)	*n* (%)	*n* (%)	*n* (%)
Low risk	94 (66.7)	107 (75.9)	135 (95.7)	77 (53.5)
Moderate-high risk	47 (33.3)	34 (24.1)	6 (4.3)	67 (46.5)
Concordance study		Dorica	Regicor	Score
		*K* (*p*)	*K* (*p*)	*K* (*p*)
	Framingham	0.709 (<0.001)	0.163 (<0.001)	0.464 (<0.001)
	Dorica		0.245 (<0.001)	0.296 (<0.001)
	Regicor			0.066 (0.067)

K: kappa concordance index; *p*: *p* value.

**Table 4:** Characteristics of the general population and rheumatic disease cases (RA and PsA).

	General population *n*=1844	Rheumatoid arthritis, *n*=75 (49.7%)		Psoriatic arthritis, *n*=76 (50.3%)	
	Mean ± SD	Mean ± SD	*p* ^1^	Mean ± SD	*p* ^2^
BMI (kg/m^2^)	29.2 ± 4.7	27.7 ± 4.8	**0.006**	28.3 ± 5.3	0.103
Adjusted Charlson index	2.2 ± 1.8	3.1 ± 1.5	**<0.001**	2.5 ± 1.4	0.630
Abdominal perimeter (cm)	95.5 ± 12.7	97.6 ± 13.0	0.160	98.1 ± 14.7	0.082
LDL	132.0 ± 31.4	121.8 ± 33.7	**0.006**	122.5 ± 30.9	**0.009**
Ankle-brachial index	1.2 ± 3.5	1.2 ± 0.1	0.999	1.2 ± 0.1	0.999
	*n* (%)	*n* (%)	*p* ^1^	*n* (%)	*p* ^2^
Charlson index					
PAD	74(4.1)	1(1.3)	0.384	0(0.0)	0.139
Cerebrovascular disease	74(4.1)	1(1.3)	0.384	0(0.0)	0.139
Heart failure	31(1.7)	1(1.3)	0.818	1(1.3)	0.831
AMI	87(4.8)	2(2.7)	0.583	1(1.3)	0.267
Diabetes	244 (13.4)	7 (9.3)	0.326	7 (9.2)	0.308
Without organ damage	217(11.9)	5(6.7)	0.242	6(7.9)	0.395
With organ injuries	27(1.5)	2(2.7)	0.723	1(1.3)	0.702
Arterial hypertension	663(36.5)	26(34.7)	0.916	33(43.4)	0.228
Smoking habit					
Nonsmoker	1008(55.0)	36(48)	0.308	37(48.7)	0.363
Ex-smoker	505(27.6)	29(38.7)	**0.044**	19(25)	0.744
Smoker	320(17.5)	10(13.3)	0.454	20(26.3)	**0.064**

BMI: body mass index; LDL: low-density lipoprotein; PAD: peripheral arterial disease; AMI: acute myocardial infarction; ^1^*p* value for contrasts general population vs RA; ^2^*p* value for contrasts general population vs PsA.

## Data Availability

The data used to support the findings of this study are restricted by the Clinical Research Ethics Committee of Galicia in order to protect patient privacy. Data are available from Cristina González-Martín, Research Group in Clinical Epidemiology, Department of Health Sciences, Faculty of Nursing and Podiatry, University of A Coruña (UDC), Ferrol Campus, Ferrol, Spain, for researchers who meet the criteria for access to confidential data.
